# Laceration of Perineum in Forceps Cases

**Published:** 1863-08

**Authors:** C. Ricketts


Laceration of Perineum in Forceps Cases.—*Prevention of.*—The adoption of the following plan in forceps cases obviates all danger of lacerating the perineum. When the forceps have been applied, and the head of the child has been brought down sufficiently low on the perineum that the fore-finger of the accoucheur introduced into the rectum can reach, for instance, in occipito-anterior presentations, beyond the frontal eminence, the instrument should be withdrawn. or, if that be not convenient, all traction should be discontinued, and the finger, still retained in the rectum, should be used after the manner of the vectis, pressure being made with it, first on the space between the frontal eminences and the root of the nose, and afterwards on the upper jaw, and perhaps, if necessary, on the chin, as these parts successively come within reach. By this means the head is made to rotate in the axis of the outlet, and, being directed forwards under the pubes, its pressure on the perineum is greatly relieved.—(Dr. C. Ricketts.) —*Id.*

